# Engineering ex vivo T-cell expansion for CAR-T therapy: bridging biological fidelity and bioprocess scalability through platforms, functional consumables, and integrated closed manufacturing

**DOI:** 10.3389/fimmu.2026.1981337

**Published:** 2026-10-20

**Authors:** Qingzhi Wu, Menghui Jiang, Yuanyuan Li, Hui Huo, Haoyuan Cheng, Tian Jia, Yushu Wang, Ziwei Wang, Mengdie Ji, Jingjing Lin, Deyue Jiang, Nuo Zhang, Fuliang Chen

**Affiliations:** Anhui Provincial Key Laboratory of Infection and Immunity, School of Basic Medical Sciences Bengbu Medical University, Bengbu, Anhui, China

**Keywords:** automated manufacturing, bioreactor, CAR-T cell therapy, cell therapy, culture consumables, ex vivo T-cell expansion, single-use system

## Abstract

**Background:**

Chimeric antigen receptor T (CAR-T) cell therapy has changed the treatment of relapsed or refractory B-cell malignancies and multiple myeloma, but manufacturing time, cost, variability, and access remain major barriers.

**Scope:**

In this critical narrative review, we position ex vivo T-cell expansion at the interface of biological signaling and engineering design and dissect how each unit operation—from selection and activation to harvest and formulation—influences the final therapeutic product.

**Key advances:**

Static gas-permeable culture, rocking-motion and stirred-tank bioreactors, hollow-fiber and perfusion systems, biomaterial scaffolds, microcarriers, and defined activation nanoparticles offer distinct routes to scalable expansion. Closed automated platforms now integrate multiple unit operations, while microfluidic systems, rapid manufacturing, and process analytical technologies (PAT) create new possibilities for monitoring and control, with data-driven and AI-assisted prediction emerging as supporting tools rather than established capabilities.

**Challenges:**

Expansion remains constrained by the trade-off between cell number and preservation of memory phenotype, metabolic fitness, and potency. Donor variability, raw-material quality, consumable dependence, limited interoperability, and incomplete potency assays complicate standardization.

**Outlook:**

Future progress will depend on coordinated development of defined raw materials, functionally designed consumables, closed-system engineering, interoperable automation, and data-driven process control. The goal is not simply to expand more cells, but to reproducibly manufacture the right cells at an acceptable cost.

## 1 Introduction

CAR-T cell therapy has moved from an experimental form of adoptive immunotherapy to an established treatment modality for several relapsed or refractory hematologic malignancies (1). Pivotal studies of CD19-directed products demonstrated durable responses in B-cell acute lymphoblastic leukemia and aggressive B-cell lymphoma (2–4), and BCMA-directed products subsequently extended the clinical reach of CAR-T therapy to multiple myeloma (5, 6). Lisocabtagene maraleucel and other products have further diversified the available product landscape (7). Recent reviews continue to emphasize both the therapeutic impact of these products and the practical barriers that limit their timely delivery (8).

These barriers are not incidental but central to the clinical success of CAR-T therapy; in a very real sense, they are part of the therapy itself. Autologous CAR-T production begins with leukapheresis and proceeds through cell selection, activation, gene transfer, expansion, harvest, formulation, quality control, and release. Each step must preserve cell identity and function while maintaining sterility and traceability. Manufacturing reviews consistently identify expansion as a key determinant of product yield, phenotype, process duration, labor intensity, and cost (9–12). At the same time, process choices made for operational convenience can alter memory differentiation, exhaustion, metabolism, and *in vivo* persistence (13–15).

This dual mandate defines the core manufacturing paradox: achieving the numerical threshold for a clinical dose must not come at the expense of the phenotypic and functional attributes that underpin therapeutic efficacy. Highly expanded products may contain abundant effector cells yet show reduced stem-like or central memory features, increased inhibitory receptor expression, or impaired metabolic fitness. Conversely, conditions that preserve early memory phenotypes may reduce fold expansion or complicate scale-up. This quantity–quality trade-off explains why expansion technologies cannot be evaluated by fold growth alone (15, 16).

Efforts to solve this problem have followed several overlapping routes. Gas-permeable static systems improve oxygen delivery with minimal manipulation. Rocking-motion and stirred-tank bioreactors introduce controlled mixing and monitoring. Hollow-fiber and perfusion systems intensify nutrient exchange. Biomaterial scaffolds, microcarriers, artificial antigen-presenting cells, and nanoparticle reagents seek to reproduce selected features of physiological T-cell activation. Closed automated instruments integrate multiple unit operations into a single sterile flow path (17–19). Regulatory and quality frameworks add another layer of constraint because devices, consumables, raw materials, and process analytics must be validated as parts of a complete medicinal product manufacturing system (20, 21).

This review focuses on ex vivo suspension expansion of T cells for CAR-T manufacturing. It does not attempt to cover *in vivo* expansion strategies, feeder-cell-dependent protocols, or non-T-cell immune cell products except where they clarify a manufacturing principle. The discussion is organized around three questions: how culture conditions shape T-cell fate, which expansion platforms and consumables best support scalable production, and how integrated automation can make CAR-T manufacturing more reproducible and accessible. Notably, it remains debated whether a ‘one-size-fits-all’ expansion platform is even desirable, given the inherent biological heterogeneity of patient-derived starting materials—a question to which we return in Sections 3 and 7.

Literature search and scope. This article is a critical narrative review, and its conclusions are based on a structured but non-systematic survey of the literature. Relevant publications were identified through searches of PubMed, Scopus, and Web of Science for English-language articles published between January 2016 and February 2026, using combinations of the terms ‘CAR-T’, ‘T-cell expansion’, ‘bioreactor’, ‘cell therapy manufacturing’, ‘activation reagent’, ‘single-use’, ‘closed system’, ‘gene transfer’, ‘cryopreservation’, ‘potency assay’, and ‘automation’. Earlier landmark studies were included where they established foundational methods or mechanisms. We prioritized primary process studies, head-to-head platform comparisons, clinical manufacturing reports, and regulatory documents; conference abstracts and non-peer-reviewed preprints were excluded. Because the field lacks standardized reporting of expansion metrics, no formal meta-analysis was attempted, and the quantitative values summarized in **Table 1** are presented as indicative ranges rather than pooled estimates.

**Table 1:** Indicative quantitative anchors for representative expansion platforms.

Platform	Typical working volume	Achievable viable cell density	Representative fold expansion (duration)	Typical seeding density	Indicative consumable cost band	Key sources
G-Rex (static, gas-permeable)	≈0.3–1 L per vessel; scale-out by vessel number	≈0.5–5 × 10^6 cells/mL	≈30–100-fold over 7–14 days	≈0.3–1 × 10^6 cells/mL	Low	Bajgain et al. (22)Gagliardi et al. (23)Gotti et al. (24)
Xuri/WAVE (rocking motion)	0.1–25 L bags	≈1–10 × 10^6 cells/mL (higher with perfusion)	≈20–100-fold over 7–14 days	≈0.2–1 × 10^6 cells/mL	Moderate–high	Somerville et al. (25)Smith (26)
Automated stirred-tank	≈0.25–3 L (development scale); conventional scale-up beyond	≈2–5 × 10^6 cells/mL in batch; up to ≈1.5 × 10^7 cells/mL with perfusion	≈20–80-fold over 6–12 days	≈0.3–1 × 10^6 cells/mL	Moderate	Costariol et al. (27), 28); Hood et al. (29)Springuel et al. (30)
Hollow-fiber/perfusion (e.g., Quantum)	≈50–600 mL single-use circuit	High local density; total yield ≈1–6 × 10^9 cells	≈50–200-fold over 7–9 days, including low-seed processes	Low total seed (≈10^7 cells)	Moderate–high	Coeshott et al. (31)Marshall et al. (32)
CliniMACS Prodigy (integrated)	≈70–250 mL culture chamber	≈1–3 × 10^6 cells/mL	≈10–60-fold over 8–12 days	≈0.5–2 × 10^6 cells/mL	High	Mock et al. (33)Zhu et al. (34)
Cocoon (integrated)	≈50–200 mL cassette	≈1–5 × 10^6 cells/mL	≈10–50-fold over 7–10 days	Platform-defined	High	Trainor et al. (35)
Microfluidic bioreactors	μL–mL scale	High-density, research scale	Not yet GMP-relevant	Device-specific	Not established	Sin et al. (36)Markelov et al. (37)

Values are ranges compiled from the cited primary reports and are intended to support orientation rather than direct cross-platform ranking; actual performance varies with donor material, activation reagent, cytokine strategy, and product type. Cost bands are relative and indicative: low denotes commodity bags and films with few proprietary parts, moderate denotes platform-specific single-use sets, and high denotes proprietary closed cassettes or tubing sets with dedicated reagent kits; absolute costs vary by region, volume, and contracting.

Several recent reviews have surveyed CAR-T manufacturing platforms, process parameters, and automation strategies (9–14). The present review is intended to complement rather than duplicate them, and three features distinguish our approach. First, we treat ex vivo expansion as a cell-fate decision rather than as a production step, and we use this framing to organize the comparison of platforms; the operative question is not which device achieves the highest fold expansion, but which combination of physical, chemical, and temporal conditions preserves a defined functional phenotype. Second, we integrate mechanoimmunology, oxygen and metabolic signalling, and material science into a single process framework, so that platform physics, consumable chemistry, and T-cell biology are assessed against the same set of critical quality attributes instead of being discussed in separate literatures. Third, we deliberately place biological and engineering criteria alongside cost, supply-chain, and regulatory constraints, and we argue that the quantity–quality trade-off, rather than any single technology gap, is the organizing problem of the field. The resulting framework is intended to support process selection, consumable design, and technology-transfer decisions rather than simply to catalogue the available technologies. **Table 2** makes this positioning explicit by comparing the coverage of the seven reviews cited above with that of the present review.

**Table 2:** Positioning of the present review relative to recent CAR-T manufacturing reviews.

Review	Scope focus	Expansion treated as a cell-fate decision	Quantitative platform anchors	Consumable and material specification	Cost and supply-chain analysis	Current regulatory framework
Vormittag et al. (9)	End-to-end manufacturing guide	Partial	No	Partial	Partial	No
Abou-El-Enein et al. (10)	Scalable manufacturing and process bottlenecks	Partial	No	Partial	Partial	Partial
Song et al. (12)	Scale-up and scale-out strategies	Partial	Partial	No	Partial	No
Ayala Ceja et al. (11)	Process parameters and next-generation strategies	Partial	No	Partial	Partial	Partial
Ramamurthy et al. (13)	Manufacturing advances, including materials	Partial	No	Partial	Partial	No
Agliardi et al. (14)	Accelerating and optimizing manufacture	Partial	No	No	Partial	Partial
Rafei et al. (8)	Clinical development and future prospects	No	No	No	Partial	Partial
Present review	Expansion as a cell-fate decision across platforms, consumables, and closed integration	Yes (organizing frame)	Yes (**Table 1**)	Yes (Section 4)	Yes (Section 6.2)	Yes (Section 6.3)

‘Yes’ indicates treatment as an organizing theme with dedicated analysis; ‘Partial’ indicates mention without dedicated analysis; ‘No’ indicates absence. The comparison is intended to make the incremental contribution of this review explicit rather than to rank the cited works.

## 2 Biological logic of ex vivo T-cell expansion

### 2.1 Expansion output is a cell-fate decision, not only a growth rate

The clinical performance of CAR-T cells is influenced by the differentiation state of the infused product. Analyses of responding patients have associated effective therapy with less differentiated, memory-like T-cell populations, whereas terminal differentiation and exhaustion are generally associated with poorer persistence (38, 39). The distinction matters because ex vivo expansion can inadvertently drive the very phenotypes that a therapeutic product should avoid. Strong or sustained TCR stimulation, tonic CAR signaling, high cytokine exposure, and prolonged culture can promote effector differentiation and exhaustion (40–42). Tonic signaling provides a concrete mechanistic example. Ligand-independent clustering of the CAR at the cell surface sustains basal CD3ζ phosphorylation, so the receptor continues to signal in the absence of antigen. This low-grade but unremitting signal drives sustained NF-κB and AP-1 activity, raises the expression of inhibitory receptors such as PD-1, TIM-3, and LAG-3, and progressively restricts proliferative capacity and cytokine secretion (40). Exhaustion can therefore be programmed into the product during the earliest days of culture, well before any decline in viability becomes apparent. In contrast, preservation of T memory stem cell-like and central memory populations is commonly associated with improved proliferative reserve and persistence (43).

Expansion platforms therefore need to be judged by a composite output. Useful measures include viable cell number and transgene expression. They also include CD4/CD8 composition, memory phenotype, and exhaustion markers. Cytokine production, cytotoxicity, metabolic state, and process safety complete the set. Direct platform comparisons support this view. Expansion systems can yield distinct differentiation states even when starting material and culture duration are similar (16).

### 2.2 Activation strength, duration, and spatial organization

T-cell activation requires coordinated T-cell receptor (TCR) and costimulatory signaling. In clinical manufacturing, anti-CD3/CD28 beads, immobilized antibodies, soluble polymeric reagents, and biomimetic scaffolds all attempt to provide this signal in a controllable form. Signal dose is not synonymous with signal quality. Bead size, ligand density, ligand orientation, receptor clustering, contact duration, and the mechanical properties of the presenting surface can change the balance between proliferation, differentiation, and activation-induced dysfunction (44–46).

Mechanobiology has emerged as an equally critical, yet often overlooked, regulatory layer of this control. T-cell activation involves force generation and cytoskeletal remodeling, and the physical context in which ligands are presented can influence signaling (47). Reviews of CAR-T mechanoimmunology argue that stiffness, viscoelasticity, confinement, and fluid shear should be treated as process parameters rather than background conditions (48). More recent work shows that β2-integrin-mediated mechanical signaling can decouple proliferation from differentiation and support stem cell-like CAR-T products (49). These findings do not imply that every mechanical intervention is beneficial; rather, they indicate that mechanical cues should be measured, controlled, and linked to defined quality attributes.

### 2.3 Oxygen, pH, and metabolic reprogramming

Activated T cells undergo rapid metabolic reprogramming, with increased glycolytic flux supporting biomass production and effector function (50, 51). Excessive or poorly controlled glycolytic commitment, however, can favor terminal effector differentiation, whereas interventions that restrain glycolysis have been shown to enhance CD8+ T-cell memory and antitumor activity in preclinical models (52). Manufacturing conditions can push cells along these trajectories. Oxygen availability, culture pH, nutrient concentration, and waste accumulation are therefore not merely engineering variables; they are inputs into T-cell fate.

Process studies have shown that dissolved oxygen and pH can influence CAR-T-cell expansion, differentiation, and function (53, 54). Crucially, the oxygen tension encountered during the initial activation phase can epigenetically imprint a persistent functional state that shapes subsequent persistence and therapeutic efficacy (55). In practical terms, this means that the oxygen tension present during the first days of activation leaves stable epigenetic marks, such as DNA methylation and histone modification patterns, on genes that control memory and exhaustion programmes. Because these marks persist after the cells are returned to standard culture conditions, they translate into measurable downstream consequences: altered expression of TCF7, CCR7, and PD-1, a lower proportion of T memory stem cell-like cells in the final product, shorter *in vivo* persistence after adoptive transfer, and reduced antitumor efficacy in preclinical models (55). These observations help explain why gas-permeable bags, rocking bioreactors, stirred vessels, and perfusion systems can produce different products even when nominal medium formulations are identical. Poor mass transfer may create oxygen gradients, local acidification, lactate accumulation, or nutrient depletion, while aggressive mixing may improve homogeneity at the cost of mechanical stress. The relevant objective is not maximal oxygenation or maximal mixing, but a stable microenvironment matched to the metabolic demands of the cells.

### 2.4 A useful process framework

Across these mechanisms, ex vivo expansion can be viewed as a chain linking external interventions to cellular outputs. Process parameters and material properties alter oxygen tension, shear, activation-signal organization, nutrient availability, and waste clearance. Those microenvironmental features then engage TCR signaling, oxygen-sensing pathways, mechanotransduction, and metabolic control, ultimately shaping proliferation, memory differentiation, exhaustion, and potency. This framework is useful because it prevents a purely equipment-centered comparison. A rocking bag, a gas-permeable flask, a stirred vessel, and a biomaterial scaffold may all act on different parts of the same biological network, but none is intrinsically superior outside a defined product and process context.

## 3 Expansion platforms

### 3.1 Static culture and gas-permeable systems

Conventional plates and T-flasks remain useful for early method development because they are inexpensive, widely available, and compatible with small-volume screening. Their limitations become clear as the process moves toward clinical manufacturing: open handling increases contamination risk, manual feeding and sampling are labor-intensive, and headspace-based gas exchange can create gradients at higher cell densities. Gas-permeable culture systems were developed to address part of this problem. Early work with tumor-infiltrating lymphocytes showed that gas-permeable flasks could support clinically relevant expansion with simplified handling (56). The G-Rex platform subsequently demonstrated that a large gas-permeable surface can support high-density suspension culture without continuous agitation (22, 24).

A practical advantage of static gas-permeable systems is operational simplicity. Cells remain undisturbed while oxygen and carbon dioxide exchange through the membrane. Closed-system workflows have also integrated activation, gene transfer, and expansion in G-Rex devices, illustrating how a static platform can reduce transfers and support clinical-grade processing (23). Nevertheless, static systems do not eliminate scale-out constraints. Multiple vessels may be required for high-dose or multi-patient production, online monitoring is limited, and final harvest may still require concentration, washing, and formulation steps outside the culture device.

### 3.2 Rocking-motion bioreactors

Rocking-motion systems, including WAVE and Xuri platforms, use a gently moving culture bag to create a thin liquid layer and repeated surface renewal. This geometry improves gas exchange and mixing while avoiding the impeller-associated shear of a stirred vessel. Clinical-scale lymphocyte expansion in the WAVE bioreactor was reported more than a decade ago (25), and Xuri-based protocols remain widely used for CAR-T expansion (26). Broader reviews of bioreactor systems identify rocking platforms as a mature option for closed, scalable T-cell culture (57, 58).

The strengths of rocking systems are practical as well as biological. Disposable bags support closure, temperature and gas control are established, and scale-up can be achieved without changing the fundamental culture principle. The main limitations are cost, platform-specific consumables, restricted sensor options in some configurations, and the possibility that mixing and oxygen transfer become less uniform as volume and cell density rise. As with all platforms, process transfer should include direct comparisons of cell phenotype and function rather than assumptions based on fold expansion. The contrast with stirred-tank systems is instructive. Rocking platforms generate mixing through periodic tilting that repeatedly renews a thin surface film, so the energy input experienced by the cell is low and the flow field is dominated by transient interfacial movement rather than by a localised impeller wake. Stirred tanks instead deliver better homogeneity and much tighter control of dissolved oxygen, pH, and feeding, but they concentrate hydrodynamic energy at the impeller tip and in the bulk turbulence it generates. The choice between the two is therefore not simply a matter of ‘gentle versus controlled’. It is a trade-off between a low-shear but weakly instrumented environment and a well-controlled environment in which shear, oxygen transfer, and nutrient gradients must all be managed at the same time.

### 3.3 Stirred-tank bioreactors

Stirred-tank systems bring conventional bioprocess control to T-cell manufacturing. They offer active mixing, online monitoring, dissolved-oxygen control, pH control, and established scale-up principles. The central concern is whether suspension T cells tolerate impeller-driven hydrodynamics without unacceptable viability loss or phenotypic change. Hydrodynamic stress here refers to the mechanical forces that a cell encounters in the flow field. These include turbulent eddies small enough to distort the cell, laminar shear in the boundary layer at the vessel wall and impeller surface, and intermittent collisions with the impeller or with gas bubbles. T cells are comparatively large, approximately 8–12 μm in diameter (27), with a deformable plasma membrane and no cell wall. Moderate shear can therefore transiently stretch the membrane, engage mechanosensitive channels and integrins, and perturb receptor–ligand bonds at the cell surface, whereas higher levels of energy dissipation may cause membrane poration, cytoskeletal disruption, and cell death. Impeller-driven systems consequently carry two distinct risks: sub-lethal mechanical signalling that may shift differentiation without reducing viability, and overt mechanical damage that reduces yield. Work in automated stirred-tank systems has shown that primary human T cells and CAR-T cells can be manufactured under controlled agitation (27, 28). These studies support feasibility, but they also show that impeller speed, seeding density, feeding strategy, and oxygen control must be optimized together.

Quality-by-design (QbD) studies are particularly informative because they treat the stirred tank as a multivariable process rather than a simple vessel. A QbD investigation of automated stirred-tank CAR-T production examined seed train duration, activation conditions, cell density, and interleukin-2 (IL-2) concentration to improve process understanding and product quality (29). More recent work has optimized perfusion feeding in serum-free medium to intensify CAR-T expansion in stirred-tank systems (30). These studies indicate that stirred tanks are not merely borrowed protein-production hardware; they can become T-cell manufacturing platforms when their control capabilities are aligned with immunological quality attributes. A contrast with monoclonal antibody manufacturing is instructive here. Decades of process optimization and cost-reduction work in that field have produced highly standardized, high-titre platform processes, in which scale-up logic and automation maturity rest on a well-defined molecular product (59). Living cell therapies cannot follow that template directly, because the product is a heterogeneous, patient-derived cell population whose quality is defined by phenotype and function rather than by titre and purity. Stirred tanks therefore bring valuable control capability to CAR-T manufacturing, but that capability has to be directed at immunological quality attributes rather than at the metrics inherited from protein production.

### 3.4 Hollow-fiber, perfusion, and three-dimensional culture systems

Hollow-fiber and perfusion systems aim to intensify culture by continuously exchanging nutrients and waste while retaining cells in a controlled compartment. The Quantum hollow-fiber system has been used for large-scale expansion and characterization of CD3+ T cells (31), and bioreactor reviews often place hollow-fiber systems between rocking bags and fully integrated closed platforms in terms of complexity and monitoring capability (57, 58). Perfusion can reduce nutrient depletion and metabolite accumulation, but it also adds pumps, sensors, tubing, and control logic that must be validated.

Three-dimensional matrices and immobilized culture systems offer a different route. Instead of relying only on suspension culture, they provide a structured environment in which cells interact with a biomaterial scaffold. Thermo-responsive matrices have been used to encapsulate and expand T and CAR-T cells *in vitro* (60). Such systems may improve local cell–material interactions or simplify recovery, but they remain less mature than bag- and reactor-based culture. Key questions include scalable fabrication, sterile filling, cell recovery, residual material clearance, and comparability of phenotype after release from the matrix.

### 3.5 Critical synthesis: matching platform physics to product biology

While each platform offers distinct operational advantages, platform selection cannot be decoupled from the biological endpoint. Static gas-permeable systems such as G-Rex are frequently described as preserving central memory phenotypes (24). That impression, however, rests largely on individual platform studies and process reports rather than on head-to-head comparisons performed under matched activation and cytokine conditions, and comparative work shows that differentiation state can differ between platforms even when starting material and culture duration are similar (16). Claims of phenotypic superiority should therefore be treated as hypothesis-generating until they are confirmed in matched comparative studies. In addition, the limited mixing capability of static systems may introduce gradients in glucose, lactate, and dissolved oxygen that are masked by conventional bulk measurements. Conversely, the robust control offered by stirred-tank systems (29) is accompanied by higher hydrodynamic stress. We treat the link between that stress and terminal differentiation as a potential rather than an established mechanism. Mechanotransduction pathways capable of influencing T-cell differentiation have been mapped (48, 49), and stirred-tank studies consistently report that agitation and oxygen transfer must be co-optimized against phenotypic readouts, but a causal relationship between impeller-derived shear and terminal differentiation has not been demonstrated directly. Testing it would require experiments in which shear is varied independently of oxygen transfer, feeding, and cell density. Rocking-motion and hollow-fiber platforms occupy intermediate positions along this control-versus-stress spectrum, each with its own monitoring blind spots. The choice of platform should therefore be dictated not by historical precedent or operational familiarity, but by a risk-based assessment of how its physical parameters impact the critical quality attributes (CQAs) of the specific CAR-T product. It is also noteworthy that, in the literature surveyed here, we did not identify a study that reports the cost per quality-adjusted CAR-T cell for these platforms in a directly comparable way; we offer this as an observation about the current evidence base rather than as a claim that no such analysis exists.

## 4 Activation materials and culture consumables

Because activation is the point at which material design most directly shapes T-cell fate, we organize this section by the dimensionality of signal presentation: reagents that deliver stimulation in suspension (Section 4.1), materials that present signals on surfaces or within structured matrices (Section 4.2), the vessels, films, and coatings that define the cell–material interface (Section 4.3), and the media and cytokines that complete the raw-material landscape (Section 4.4).

### 4.1 Suspended and soluble activation: from magnetic beads to defined nanoparticles

Anti-CD3/CD28 stimulation has a long history in clinical T-cell expansion. Early large-scale work showed that CD3/CD28 costimulation could generate clinically relevant CD4+ T-cell populations (61). Magnetic beads became a standard manufacturing reagent because they provide robust activation and can be removed by magnetic separation. These benefits are counterbalanced by well-documented logistical drawbacks: bead removal adds a unit operation, residual beads must be controlled, and high-performance reagents manufactured under good manufacturing practice (GMP) conditions are costly. Soluble polymeric activation reagents reduce the need for magnetic separation, while immobilized antibodies can provide localized stimulation without a recoverable particulate phase. Emerging nanoparticle platforms are designed to decouple potent signaling from protracted physical retention, thereby obviating the need for magnetic separation while potentially mitigating tonic signaling-induced exhaustion. Dextran-based T-cell expansion nanoparticles, for example, were developed as a removable bead-free reagent for manufacturing CAR-T cells with augmented efficacy (62). Such approaches are promising because they address a process bottleneck rather than merely increasing signal strength. Their value will depend on GMP availability, lot-to-lot consistency, clearance testing, and performance across donors and product types. For polymer-based reagents, lot-to-lot consistency should be defined by measurable attributes rather than by nominal composition. Useful metrics include polymer molecular weight and dispersity (Mw and Mw/Mn), ligand density and its distribution across particles, particle diameter and polydispersity index, surface charge (zeta potential), free residual ligand, endotoxin and bioburden, and residual solvent or cross-linker. Functional metrics matter at least as much: the CD3/CD28 signal strength delivered at a defined particle-to-cell ratio, and the expansion and phenotype obtained with a reference T-cell lot. Acceptance ranges should be derived from multi-lot data and should be shown to correlate with product critical quality attributes.

### 4.2 Surface- and matrix-dependent activation: artificial antigen-presenting cells, scaffolds, and microcarriers

Biomaterial-based activation systems attempt to reproduce selected features of antigen presentation in a defined and scalable format. Mesoporous silica scaffolds functionalized with activation cues and cytokine-releasing components enabled efficient ex vivo expansion of primary T cells (44), and a subsequent protocol described practical use of artificial antigen-presenting cell scaffolds for human T-cell activation and expansion (63). Viscoelastic synthetic antigen-presenting cells have since shown that matrix mechanics can influence the potency of the expanded product (46). Soft materials can also improve T-cell expansion compared with rigid presentation surfaces (45), and biodegradable nanoellipsoidal artificial antigen-presenting cells have been explored for antigen-specific activation (64).

Microcarrier- and hydrogel-based systems extend these ideas into three dimensions. Hydrogel microtubes have been used for automated expansion of primary human T cells in a scalable and cell-friendly format (65). Biomaterials reviews emphasize that composition, degradation, stiffness, ligand density, and release kinetics must all be controlled if such systems are to support clinical manufacturing (66). The main challenge is translation. A material that performs well in a laboratory assay must still be manufactured reproducibly, sterilized without loss of function, filled into a closed process, separated from the final cells, and shown not to introduce unacceptable residuals.

### 4.3 Culture vessels, multilayer films, and functional coatings

Culture vessels and bags are often treated as passive containers, but their materials and surfaces influence process performance. Polystyrene plates and flasks differ in wettability and protein adsorption depending on surface treatment. Wettability controls this behaviour because it sets the interfacial energy between the polymer surface and the aqueous medium. On a hydrophilic surface, water is strongly bound in an organized layer that proteins must displace, so adsorption is weaker and more reversible, and the adsorbed protein tends to retain a near-native conformation. On a hydrophobic surface, dehydration of the interface is energetically favourable, and proteins such as albumin, fibronectin, and immunoglobulins adsorb rapidly and often irreversibly, frequently with partial unfolding that exposes new epitopes and changes subsequent cell adhesion. Surface chemistry therefore acts as an upstream process parameter: it determines which proteins coat the vessel, at what density and in what orientation, and hence which adhesion and activation signals the T cell actually encounters (66, 67).

Bag films combine a fluid-contact layer, structural layers, tie layers, and—depending on the application—either gas-permeable layers that sustain culture respiration or barrier layers that protect stored or shipped product; the exact composition is product-specific and should not be assumed to be identical across suppliers. Gas permeability affects oxygen delivery and carbon dioxide removal, while the fluid-contact layer affects protein adsorption, cell compatibility, leachables, and particle formation. As illustrated in **Figure 1**, a representative closed single-use culture bag integrates sterile feed and sampling interfaces with a multilayer co-extruded film; within this architecture, the gas-exchange layer is decisive for sustaining oxygen delivery and carbon dioxide removal—and hence for stabilizing culture pH—while the fluid-contact layer governs protein adsorption and cell compatibility (13, 66).

**Figure 1:**
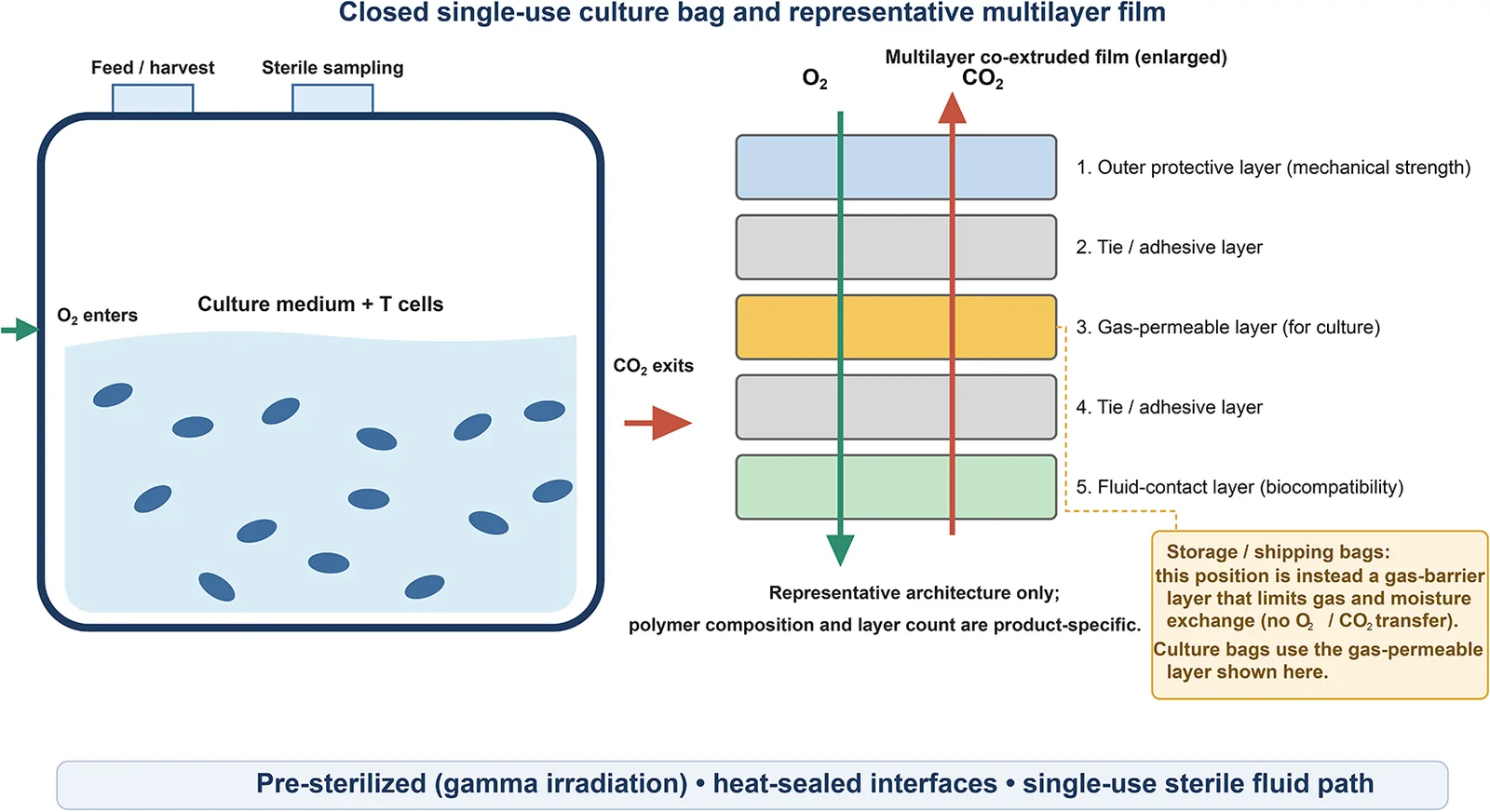
Closed single-use culture bag and representative multilayer co-extruded film. The schematic links material attributes to process behaviour and to product quality attributes. The fluid-contact layer sets surface wettability and protein adsorption, and therefore the density and orientation of adsorbed adhesion and activation ligands, which in turn influence activation strength, memory phenotype, and expression of exhaustion markers. For culture bags, a gas-permeable layer sets oxygen transfer and carbon dioxide removal, and therefore the achievable viable cell density, the stability of culture pH, glycolytic flux, and the oxygen-dependent imprinting of T-cell state discussed in Section 2.3; for storage and shipping bags, a barrier layer is instead engineered to limit gas and moisture exchange, and the two film functions should not be conflated. The tie and adhesive layers provide the mechanical strength and integrity of the sterile barrier, and therefore govern leak and particle risk and hence process safety and batch consistency. The polymer composition and layer count vary among commercial products; the diagram is therefore illustrative rather than product-specific and should not be used to infer equivalence between suppliers.

Functional coatings can add biological activity to the culture surface. Immobilized anti-CD3/CD28 antibodies concentrate activation signals at the cell–material interface, while extracellular matrix proteins or recombinant adhesion molecules can provide supplementary adhesion and survival cues. Covalent coupling generally offers better control over ligand retention and orientation than passive adsorption, but it also increases manufacturing complexity and requires more rigorous lot-release testing. Animal-derived coatings raise additional regulatory risk, because regulators expect documented source-animal traceability, viral-inactivation validation, and transmissible spongiform encephalopathy (TSE) or bovine spongiform encephalopathy (BSE) certification for such materials, in addition to the variability and immunogenicity concerns that arise from their biological origin. Defined recombinant proteins and synthetic coatings are therefore attractive, provided that their activity can be preserved after sterilization and storage (66).

### 4.4 Media, cytokines, and raw-material definition

Medium formulation and cytokine strategy are inseparable from the expansion platform. IL-2 remains widely used, but its concentration can shift the balance between effector expansion and early memory preservation; low-dose IL-2 has been reported to favor early memory phenotypes over terminal effector differentiation in CAR-T manufacturing (68). Serum-free and xeno-free media can reduce variability and regulatory risk, and comparative studies indicate that serum-free formulations can support CAR-T expansion and function (69–71). A recent review of cytokines in CAR-T manufacturing highlights both their biological importance and their role as costly, variable raw materials that require careful control (72).

Beyond IL-2, the common γ-chain cytokines IL-7 and IL-15 have become the dominant alternative strategy, and they carry the strongest evidence for memory preservation. IL-7 and IL-15 instruct the generation of T memory stem cells from naive precursors and support expansion with less terminal differentiation than IL-2 (73), and membrane-tethered IL-15 has been shown to enrich a stem-cell memory subset and to augment antitumor activity (74). IL-21 acts differently again: compared with IL-2, it confers an opposing differentiation program, restraining terminal effector commitment while preserving proliferative potential (75). Combinations of IL-7, IL-15, and IL-21 are therefore increasingly used where the objective is a less differentiated product, although GMP-grade supply, lot consistency, and cost remain non-trivial (72).

Pharmacological interventions offer a second, complementary lever on the same trade-off. Inhibition of AKT signaling during expansion uncouples T-cell differentiation from proliferation, yielding expanded but minimally differentiated products (76). Transient treatment with the kinase inhibitor dasatinib, which pauses CAR signaling, restores functionality in exhausted CAR-T cells through epigenetic remodeling (77), and mTOR inhibition with rapamycin regulates memory CD8+ T-cell differentiation (78). These interventions are attractive because they are reversible, dose-tunable, and inexpensive relative to hardware changes; their translation depends on demonstrating complete washout, defining exposure windows, and showing that the phenotypic benefit persists after transfer to cytokine-only culture. Because cytokine strategy is the cheapest available lever on the quantity–quality trade-off, we recommend that platform comparisons (Section 3.5) always report the cytokine regimen explicitly and that head-to-head studies be performed under matched cytokine conditions before phenotypic differences are attributed to platform physics.

Single-use consumables also require quality oversight. Sterility assurance, endotoxin, particles, extractables and leachables, film integrity, connector performance, shelf life, and post-sterilization functionality all affect the manufacturing process. Gamma irradiation, ethylene oxide, and steam sterilization can change polymers differently, and a material that is nominally identical may perform differently after sterilization or aging. Consumable qualification should therefore include biological performance, not only physical specifications (13, 66). This point is particularly important when replacing an established reagent or container with a lower-cost alternative: the replacement may be chemically similar but functionally different.

It is worth stating the material–process–quality linkage explicitly, because it is the basis on which consumables should be specified and released. Material attributes act on the process through a small number of mechanisms, and each mechanism has a defined quality consequence. The wettability and protein-adsorption behaviour of the fluid-contact layer determine which adhesion and activation ligands the cell encounters, and therefore influence activation strength, memory phenotype, and exhaustion. The gas-transfer properties of the film set the achievable oxygen transfer rate and carbon dioxide removal, which in turn constrain viable cell density, glycolytic flux, and the oxygen-dependent imprinting described in Section 2.3. Leachables, particles, and residual monomers act primarily as safety attributes, but they can also depress expansion and alter cytokine profiles. Ligand density and orientation on bead, polymer, and nanoparticle reagents set the effective activation dose, which is linked to CD4/CD8 composition and differentiation state. Sterilization modality changes polymer chemistry and can shift several of these attributes at once. Material attributes should therefore be specified with acceptance ranges that are justified by these mechanisms, and each consumable lot should be released against a functional performance criterion in a defined bioassay rather than against physical specification alone (13, 44, 62, 66).

## 5 Integrated and automated manufacturing

### 5.1 Process risks and design requirements

The conventional CAR-T workflow contains repeated opportunities for contamination, cell loss, and process drift. Manual open transfers, centrifugation, sampling, medium exchanges, and offline testing interrupt the sterile boundary and create operator-dependent variability. As mapped in **Figure 2**, each manual transfer, centrifugation, sampling, and medium-exchange event breaches the sterile boundary and compounds the risk of contamination, cell loss, and batch-to-batch drift; the figure distills these vulnerabilities into the core design requirements for integrated expansion devices. These requirements include a closed sterile fluid path, automation of routine operations, inline or at-line monitoring, complete data traceability, and a design that can be validated under GMP.

**Figure 2:**
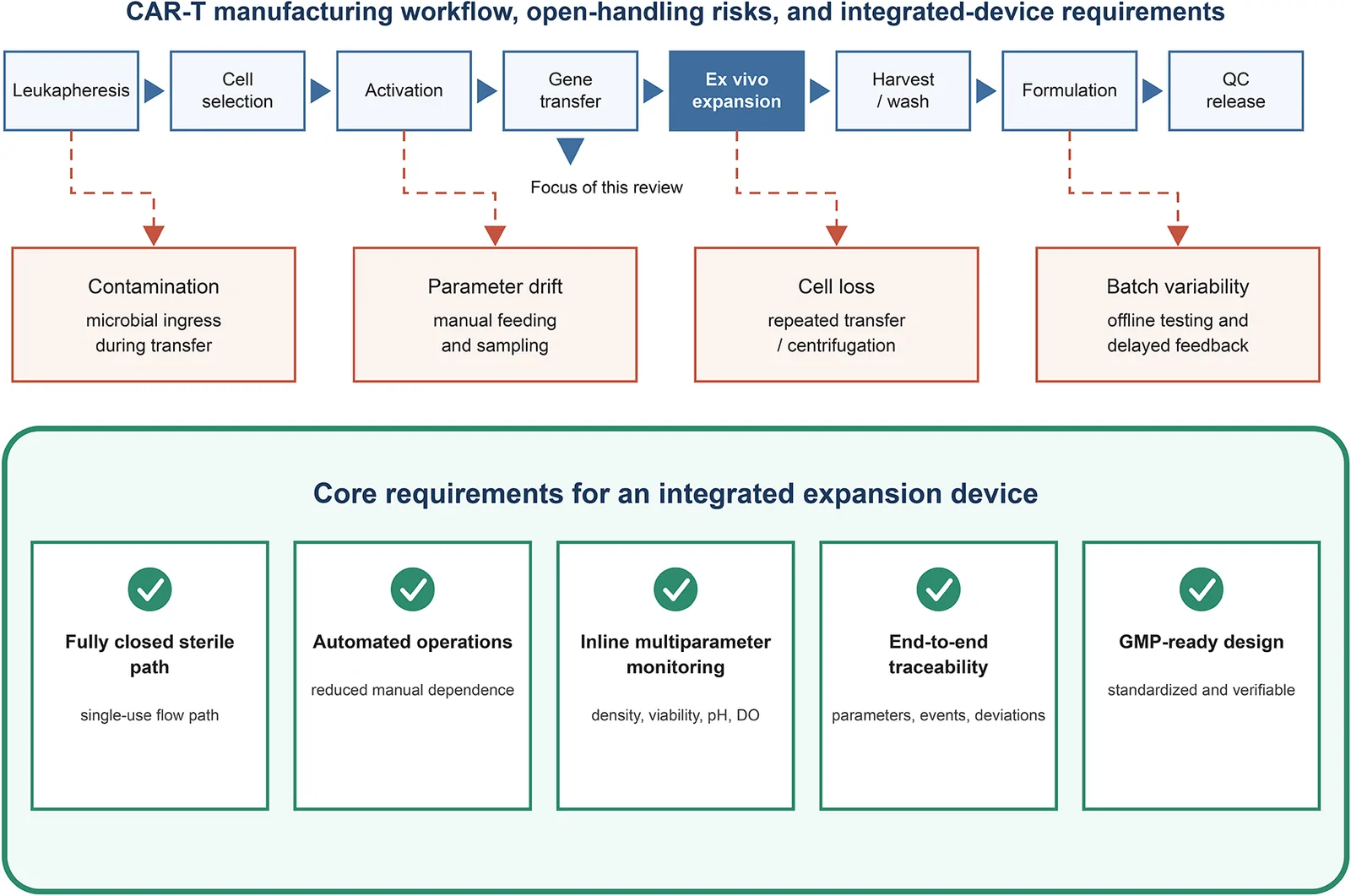
CAR-T manufacturing workflow, risks associated with open or semi-open operations, and core requirements for integrated expansion devices. Each manual or semi-open operation is mapped to the specific manufacturing failure mode it generates: open transfer to microbial ingress, manual feeding and sampling to parameter drift and loss of process knowledge, repeated transfer and centrifugation to cell loss and reduced recovery, and offline testing to delayed feedback and batch variability. The lower panel translates these failure modes into device requirements, so that each requirement can be traced to the risk it mitigates: a closed sterile fluid path protects microbiological safety, automation of routine operations supports process consistency and preservation of memory phenotype, inline multiparameter monitoring controls viable cell density and metabolism, end-to-end traceability underpins batch comparability, and GMP-ready design supports regulatory acceptability. The workflow analysis shown here corresponds to the process risks discussed in Section 5.1 and to the gene-transfer and downstream unit operations covered in Sections 5.5 and 5.6.

Automation should not be confused with simple mechanization. A device that automates individual steps but still requires manual intervention between modules may reduce labor without eliminating process risk. True integration means that cells, reagents, waste, and data move through a controlled and traceable pathway. It also means that process deviations are detected early enough to permit corrective action. Sensors for viable cell density, viability, pH, dissolved oxygen, metabolites, and pressure can support such control, but only if they remain accurate in single-use assemblies and are linked to appropriate control algorithms.

### 5.2 Commercial closed and automated platforms

Commercial systems differ substantially in how much of the workflow they integrate. CliniMACS Prodigy was shown early to support automated CAR-T manufacturing in a closed system (33), and subsequent academic-center experience demonstrated closed-system production of CD19 and dual-targeted CD20/19 CAR-T cells (34). The Cocoon platform has also been used for automated production of gene-modified CAR-T cells (35). More recently, a bioreactor with embedded control automation was used to transition an autologous T-cell process from manual to automated manufacturing (79).

These platforms illustrate two different integration philosophies. Instrument-centered systems combine several unit operations within a proprietary device and consumable set, whereas modular systems automate selected operations and connect them through closed transfers. Both can reduce open handling and operator dependence. Both can also create new dependencies on proprietary consumables, software, service, and validation packages. **Table 3** compares representative platforms by workflow coverage, monitoring capability, and major constraints; the comparison is intended to guide process selection rather than imply a universal ranking. Read in this light, **Table 2** highlights a critical dichotomy in the current automation landscape: whereas platforms such as CliniMACS Prodigy and Cocoon pursue end-to-end integration within a proprietary cassette or tubing set, modular approaches offer greater process flexibility at the expense of increased integration and validation complexity. This trade-off is not merely technical; it has profound implications for technology transfer and multi-center manufacturing strategies (80).

**Table 3:** Representative platforms for CAR-T expansion and integrated manufacturing.

Platform or approach (category)	Workflow coverage	Degree of automation	Scale-up vs scale-out strategy	Monitoring and control capability	Manufacturing maturity	Main constraints
CliniMACS Prodigy (integrated commercial platform)	Selection, activation, transduction, expansion, harvest, and formulation in one closed tubing set (33, 34)	High; end-to-end automated with user-defined programmes	Scale-out (parallel instruments)	Device-integrated process control with closed sampling; limited real-time cell-quality analytics	Clinically established; widely used in academic and commercial settings	Proprietary consumables; limited process flexibility; high capital and run cost
Lonza Cocoon (integrated commercial platform)	Automated CAR-T production in a closed cassette-based system (35)	High; cassette-based automation of core unit operations	Scale-out (one cassette per batch)	Automated unit operations with platform-specific monitoring; limited inline cell-quality sensing	Clinical and early commercial use	Cassette and reagent dependence; closed architecture limits process change
G-Rex closed workflow (expansion device)	Activation, transduction, and static expansion; harvest and downstream steps may require external systems (24, 23)	Low to moderate; manual or semi-automated closed transfers	Scale-out (multiple vessels)	Limited inline sensing; control achieved through defined feeding and handling	Widely used; clinical-grade consumables available	Gradients at high cell density; concentration and washing require additional systems
Xuri/WAVE rocking bioreactor (expansion device)	Closed suspension expansion, with optional perfusion (25, 26)	Moderate; automated temperature, gas, rocking, and perfusion control	Scale-up within bag volume, with scale-out for high dose	Temperature, gas, rocking, and perfusion control; sensor options depend on configuration	Mature clinical use in cell therapy	Consumable cost; sensor limitations; platform lock-in
Automated stirred-tank bioreactor (expansion device)	Controlled T-cell and CAR-T expansion with bioprocess-grade monitoring (27–29)	Moderate to high; automated agitation, dissolved oxygen, pH, feeding, and perfusion	Conventional scale-up using established engineering correlations	Agitation, dissolved oxygen, pH, feeding, and perfusion; mature online sensing	Established in bioprocess engineering; emerging for T-cell manufacture	Shear and optimization burden; agitation, seeding, and feeding must be co-optimized
Hollow-fiber/perfusion system (expansion device)	High-density expansion with continuous medium exchange (31, 32)	Moderate; automated perfusion and flow control	Scale-up by membrane area	Flow, pressure, and perfusion control; optional metabolite monitoring	Clinical-scale use reported, including low-seed processes	Cell recovery and process complexity; limited standardization
Modular automated systems (technology class)	Automated connection of selected unit operations (79)	Variable; determined by the modules selected	Either, depending on configuration	Embedded control and data capture across modules; often integrates legacy sensors	Process development and early clinical use	Integration and validation burden increases with module number
Microfluidic bioreactors (technology class)	Miniaturized selection, activation, culture, and analytical functions (36, 37)	High within a single device; narrow workflow coverage	Scale-out by parallelization; scale-up not yet demonstrated	High-resolution environmental and single-cell measurement potential	Early stage; limited GMP maturity	Scale-up, long-term culture stability, and GMP validation remain immature

Systems are grouped into integrated commercial platforms, expansion devices, and broader technology classes, and are compared using a consistent set of attributes: workflow coverage, degree of automation, scale-up versus scale-out strategy, monitoring and control capability, manufacturing maturity, and principal constraints. ‘Manufacturing maturity’ is defined here as a composite of (i) documented clinical-scale use under GMP, (ii) availability of GMP-grade consumables and technical support, (iii) use in marketed or late-stage clinical products, and (iv) published process-validation data; the comparison is intended to guide process selection rather than to imply a universal ranking. All works cited in this table are listed in full in the reference list, and the citation details have been checked against the final bibliography.

Because qualitative comparison alone cannot support process selection, **Table 1** adds quantitative anchors for the principal platforms for which quantitative data are available: typical working volume, achievable viable cell density, representative fold expansion and process duration, seeding density, and an indicative consumable cost band. The values are ranges compiled from the cited reports rather than guarantees, and they should be read together with the biological trade-offs discussed in Sections 2 and 3.

### 5.3 Closed-loop control and smart manufacturing

**Figure 3** presents a generalized closed-loop architecture for automated CAR-T production. Unit operations are connected through a sterile single-use assembly; inline sensors measure pH, dissolved oxygen, temperature, and pressure, whereas cell density, viability, and metabolites are generally measured at-line on sampled aliquots; the control platform adjusts feeding, gas exchange, temperature, agitation, or perfusion in response to measured deviations. This architecture resembles conventional process analytical technology (PAT), but the critical quality attributes are cellular and functional rather than purely biochemical.

**Figure 3:**
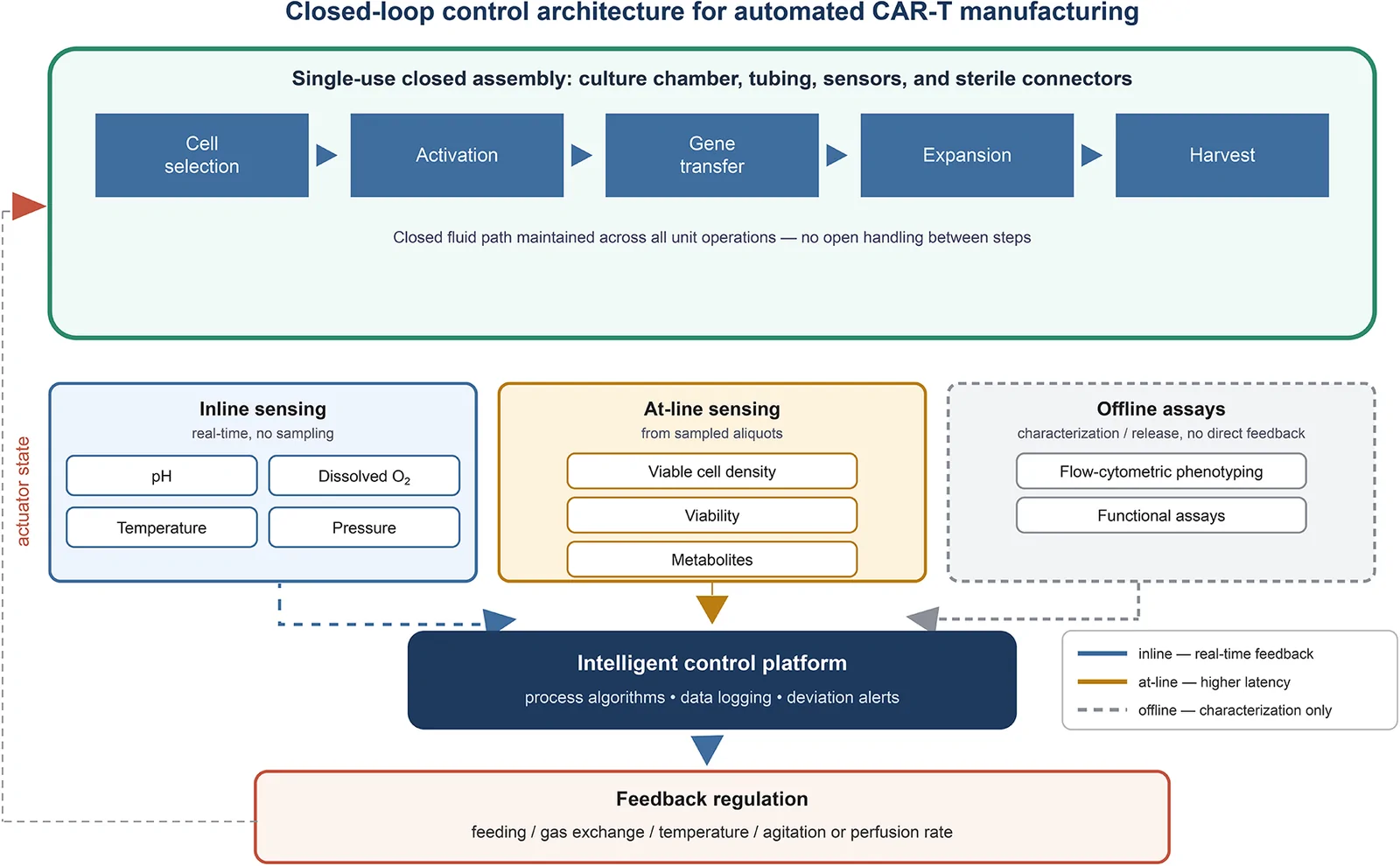
Generalized closed-loop control architecture for automated CAR-T manufacturing. A single-use assembly connects selection, activation, gene transfer, expansion, and harvest while sensors report to a control platform at three levels of integration: inline (pH, dissolved oxygen, temperature, and pressure), at-line (viable cell density, viability, and metabolites measured on sampled aliquots), and offline (flow-cytometric phenotyping and functional assays). Feedback regulation adjusts feeding, gas exchange, temperature, agitation, or perfusion to maintain the process within a defined operating range. The architecture shown here corresponds to the closed-loop control system discussed in Section 5.3.

Closed-loop control is likely to remain difficult without better real-time indicators of T-cell quality. Cell count and viability are necessary but insufficient. Microfluidic technologies offer one route to richer measurement because they can combine small sample volumes with high-content physical or functional profiling (81, 82). A high-density microfluidic bioreactor has been reported for automated CAR-T manufacturing (36), and ultrafast microfluidic production concepts are being explored (37). Cell trajectory modulation has also been proposed as a rapid biophysical profiling method for CAR-T functional phenotypes (83). In practice, cell trajectory modulation works by flowing a cell suspension through a microfluidic channel whose geometry and flow profile impose a defined combination of shear and extensional stress on each cell. Cells that differ in membrane deformability, cytoskeletal organization, and nuclear-to-cytoplasmic ratio follow measurably different paths and arrive at different downstream positions, so the trajectory itself becomes a label-free readout of biophysical state. Because the measurement takes minutes, requires only a small number of cells, and needs no staining or lysis, the same principle can be used to sort subpopulations or to flag a shift in the biophysical distribution of a culture before conventional phenotypic markers change. Its main limitations are that the trajectory is an indirect correlate rather than a direct measure of function, and that its relationship to potency-relevant outcomes still has to be established for each product. These methods are not yet substitutes for validated potency assays, but they may become process analytical tools that predict drift before a batch fails.

### 5.4 Rapid, low-seed, and point-of-care manufacturing

Rapid manufacturing challenges the assumption that extensive ex vivo expansion is always necessary. Non-activated or minimally cultured CAR-T processes can preserve less differentiated phenotypes and shorten vein-to-vein time (84). YTB323, developed with a short manufacturing process designed to preserve T-cell stemness, showed encouraging preclinical and early clinical performance (85). A next-day anti-CD19 CAR-T product has also entered first-in-human testing (86). Related work has reported low-seed CAR-T manufacture in a GMP-grade hollow-fiber platform (32), scalable CAR-T production from whole blood (87), and instant manufacturing of a BCMA-BBZ-OX40 product in multiple myeloma (88).

These approaches reduce culture time, medium use, labor, and some facility requirements. They do not remove the need for process control. Low-seed processes may be especially sensitive to starting-material composition, activation timing, transduction efficiency, and release testing. Short processes also compress the time available for sterility and potency testing below the incubation periods required by compendial growth-based methods (USP <71>; Ph. Eur. 2.6.27), increasing the value of rapid microbiological methods, risk-based release strategies, and validated inline analytics within a formal potency-assurance strategy (111). Rapid manufacturing should therefore be understood as a redesign of the entire control strategy, not simply as the omission of expansion days.

### 5.5 Gene transfer: timing, vector dose, and non-viral alternatives

Gene transfer is the unit operation most tightly coupled to expansion, yet it is often treated as a fixed step rather than as a controllable process variable. Transduction timing relative to activation materially shapes both efficiency and product phenotype: γ-retroviral vectors require dividing cells and are therefore applied after activation, whereas lentiviral vectors can transduce less activated cells and enable earlier gene transfer. Shortening the interval between activation, transduction, and harvest preserves less differentiated phenotypes; reducing ex vivo culture time improves the antileukemic activity of CAR-T cells in preclinical models (89), and minimally cultured or non-activated processes extend this principle toward the clinic (84).

Multiplicity of infection (MOI) is the second major variable. Higher MOI raises transduction efficiency but also vector copy number, with consequences for insertional mutagenesis risk, tonic signaling, regulatory expectations, and vector cost—vector is among the most expensive raw materials in the process. The vector platform itself is a strategic choice. Lentiviral and γ-retroviral systems dominate approved products, but non-viral approaches are advancing: transposon systems such as Sleeping Beauty have generated CD19-specific CAR-T cells in early-phase trials (90), and CRISPR-based genome editing has been used clinically to redirect and reprogram T cells (91). Non-viral delivery reduces vector cost and manufacturing lead time but currently demands more process development to reach comparable efficiency and viability.

For integrated platforms, the practical implication is that transduction parameters should be specified and monitored with the same rigor as expansion parameters. Transduction efficiency, vector copy number, and the phenotype of the transduced population should be treated as critical quality attributes; the timing and MOI of gene transfer should be reported in platform comparisons; and closed platforms should be qualified for a defined vector type, because tubing geometry, cell concentration at transduction, and enhancer use all influence the outcome.

### 5.6 Harvest, washing, and formulation

Expansion ends with three operations that determine whether the cultured population reaches the patient intact: harvest, washing, and formulation. As noted in Section 3.1, static systems such as G-Rex require external concentration and washing, and even integrated platforms differ in how completely they automate these steps. Counter-flow centrifugation devices—including the Sepax, LOVO, and Rotea systems—can concentrate and wash cells in a functionally closed manner, exchanging culture medium for formulation buffer while minimizing cell loss (9, 10). Manual centrifugation and open transfers, by contrast, reintroduce the contamination and variability risks that closed expansion was designed to eliminate.

These steps are not biologically neutral. Centrifugal and fluid-mechanical stress during concentration can reduce viability and recovery, particularly for cells already stressed by high-density culture; wash cycles remove residual cytokines, activation reagents, and metabolites but can also damage or lose cells; and formulation determines the interval between harvest and infusion or cryopreservation. Recovery, viability, residual-reagent clearance, and container-closure integrity should therefore be specified as process outputs, and harvest criteria should be defined by phenotype and potency-relevant markers rather than by cell count alone (11).

Integration strategy follows from this. Fully integrated instruments automate harvest and formulation within the same closed set, reducing transfers but fixing the process within proprietary consumables; modular combinations of an expansion device with a dedicated counter-flow washer offer flexibility at the cost of additional connection and validation work. Either route must close the loop described in **Figure 2**: the unit operations that follow expansion deserve the same design attention as expansion itself.

## 6 Current challenges

### 6.1 Product variability and the quantity–quality trade-off

Donor variability remains one of the largest sources of manufacturing uncertainty. Age, disease state, prior therapy, leukapheresis composition, and starting T-cell phenotype all influence expansion and final product function. Reviews of CAR-T manufacturing variability emphasize that even a well-controlled facility cannot fully remove biological heterogeneity (92). Process intensification can amplify this problem if a platform is optimized for an ideal donor but performs poorly with heavily pretreated or low-cellularity starting material.

A second challenge is the persistence of fold expansion as a dominant process metric. Historically, this metric was inherited from the first generation of adoptive cell therapies, in which tumour-infiltrating lymphocytes were expanded over weeks and the practical question was simply whether enough cells could be obtained for a therapeutic dose. Regulatory release specifications, dose-setting studies, and technology-transfer documents were built around viable cell count, and fold expansion became the common language of process development because it is easy to measure, comparable between sites, and directly tied to dose. The metric has persisted long after the biology moved on, in part because the potency endpoints that would replace it remain poorly defined and difficult to standardise (93). High fold expansion is useful only when the expanded cells retain the desired phenotype, metabolic fitness, and potency. Platform-comparison studies showing distinct differentiation states (16) and studies linking oxygen exposure to persistence (55) reinforce the need to define quality targets before optimizing scale. Fold expansion alone is therefore a poor surrogate for potency. A larger population dominated by effector-memory cells may be less useful than a smaller population enriched for T memory stem cell (TSCM)-like subsets (43).

Potency assays and biomarkers remain underdeveloped relative to the complexity of cell products (93, 94). Without better links between process measurements and clinical function, closed-loop manufacturing will remain limited to controlling surrogates rather than the attributes that matter most.

Closing this gap requires candidate assays with explicit trade-offs, and we therefore summarize the leading options. Serial or repeat-stimulation assays, in which cells are rechallenged with antigen and scored for continued proliferation and cytokine output, come closest to the biology of durable responses but are slow and difficult to standardize, making them better suited to characterization than to release. Polyfunctional cytokine profiling (e.g., single-cell IFN-γ, IL-2, and TNF-α co-production) correlates with clinical outcomes in several settings and is quantitative, but it requires specialized instrumentation. Real-time kinetic cytotoxicity assays are rapid, label-free, and amenable to automation, and they are plausible release-assay candidates once acceptance criteria are linked to dose and clinical response. Flow-cytometric panels quantifying TSCM frequency and TCF7 expression offer mechanistic grounding in memory biology and use widely available hardware, but phenotype alone remains a surrogate. A pragmatic strategy is therefore a tiered potency-assurance matrix: a robust, fast assay (killing or cytokine release) for batch release, with repeat-stimulation and high-dimensional profiling reserved for characterization and comparability—an approach consistent with recent regulatory thinking on potency assurance (111, 93, 94).

The quantity–quality framing is useful, but it should not be read as implying that a single phenotypic optimum exists. What constitutes an optimal T-cell phenotype depends on at least four contextual variables. CAR design is the first: costimulatory domains differ in the differentiation programmes they favour, and 4-1BB-based receptors are generally associated with greater persistence and a less terminally differentiated phenotype than CD28-based receptors (40, 95), so the same expansion process may not be optimal for both. Disease indication is the second: proliferative demand, antigen burden, and the immunosuppressive environment differ between B-cell acute lymphoblastic leukemia, large B-cell lymphoma, and multiple myeloma, and a phenotype that is advantageous in one setting may be less appropriate in another. Starting material is the third: heavily pretreated patients, patients with prior stem cell transplantation, and paediatric donors differ in T-cell senescence, naive-cell frequency, and the ability to expand at all, so the feasible phenotypic window is partly set before any process decision is made. Manufacturing strategy is the fourth: rapid and low-seed processes deliberately trade expansion for stemness and speed, whereas conventional processes have more opportunity to shape phenotype through medium, cytokine, and oxygen control. A single target phenotype cannot therefore be specified independently of these variables. We suggest that quality targets should be defined per product and per population, and that platform and consumable selection should follow from those targets rather than precede them.

### 6.2 Cost, supply chains, and functional consumables

High manufacturing cost continues to restrict access to CAR-T therapy (96). Cost analyses show that production economics depend on facility utilization, labor, quality control, logistics, raw materials, and failure rates as well as the price of instruments (97, 98). The contrast with monoclonal antibody manufacturing is again informative. Process optimization, platform standardization, and targeted cost-reduction strategies have driven the cost of goods for antibody therapeutics to levels that autologous cell therapy cannot approach, because living cell products are manufactured in single-patient batches from intrinsically variable starting material, with limited economies of scale and no equivalent of a platform purification train (59). Decentralized manufacturing may reduce transportation and organizational complexity, but it does not automatically reduce total cost if each site operates below capacity or duplicates quality infrastructure (99, 100).

Consumables are central to this discussion. Activation reagents, serum-free media, cytokines, closed tubing sets, bags, filters, sensors, and connectors all contribute to cost and supply risk. Replacement of imported or proprietary materials with locally produced alternatives can improve resilience, but substitution must be supported by comparable biological performance, sterility assurance, extractables and leachables data, stability studies, and traceability. Adopting a lower-cost consumable that inadvertently introduces process variability or elevates the failure rate is, in economic terms, a false economy. Functional equivalence is therefore a more appropriate goal than nominal material equivalence.

### 6.3 Standardization, interoperability, and regulation

The field currently lacks standardized interfaces and interoperable performance benchmarks across devices, consumables, sensors, and software. A process developed on one platform often cannot be transferred directly to another because bag geometry, mixing, gas exchange, sampling, and control logic differ. Recent work explicitly frames process diversity as a barrier to standardized cell-therapy manufacturing workflows (80). Interoperability is not simply an engineering convenience; it affects validation burden, technology transfer, comparability, multi-center production, and regulatory review.

Quality-by-design (QbD) provides a structured route through this complexity. QbD starts with the target product profile and critical quality attributes, identifies critical material attributes and process parameters, and uses risk assessment and designed experiments to establish a control strategy (21, 29). This framework is particularly relevant to CAR-T expansion because many variables interact: starting-cell composition, activation dose, oxygen, pH, cytokines, feeding, shear, and harvest time. The goal is not to eliminate variability, which is impossible, but to understand which variability matters and to control it within a proven acceptable range.

Regulatory expectations have also moved since the foundational statements usually cited in this space (20, 21). In the United States, FDA’s draft guidance on potency assurance for cell and gene therapy products formalizes the expectation of a potency-assurance strategy that links assays to mechanism of action across the product lifecycle (111). In Europe, the revised Annex 1 of the EU GMP Guide strengthens contamination-control-strategy requirements and provides the regulatory anchor for the closed-system and single-use processing claims made throughout this review (101). ICH Q5A(R2) updates viral-safety expectations relevant to vector and raw-material qualification (102), and compendial sterility requirements (USP <71>; Ph. Eur. 2.6.27) define the baseline against which rapid microbiological methods must be validated (103, 104). These documents matter practically because the rapid-manufacturing argument of Section 5.4 turns on release timing: when manufacturing takes one to three days, growth-based compendial sterility testing becomes the rate-limiting step, and the regulatory acceptability of rapid methods and risk-based release determines whether speed translates into earlier treatment.

### 6.4 Cryopreservation of starting material and drug product

Cryopreservation is among the best-documented determinants of expansion performance, yet it is easily overlooked because it brackets rather than constitutes the expansion step. At the front end of the process, the choice between fresh and cryopreserved leukapheresis affects starting-material composition, post-thaw viability, and subsequent expansion kinetics; cryopreservation of apheresis material can alter the recovery and function of the starting T-cell population (19, 105). Fresh starting material preserves function but constrains scheduling and logistics; cryopreserved material enables centralized collection and flexible scheduling at the cost of a freeze–thaw insult whose magnitude varies between donors.

At the back end, drug-product cryopreservation determines post-thaw recovery and potency. Controlled-rate freezing, cryoprotectant (typically DMSO) concentration, storage temperature, and thaw practice all influence viable recovery, and post-thaw function—rather than viability alone—should anchor the specification (105). Cryopreserved drug product decouples manufacturing from patient scheduling and enables distribution, but it also demands validated thaw workflows at the bedside; fresh formulations avoid these issues but compress the entire logistics chain into hours.

Two implications follow for platform selection. First, expansion platforms should be qualified with the actual starting material intended for clinical use, including cryopreserved apheresis where relevant, because performance established with fresh research-grade leukapheresis may not transfer. Second, cryopreservation steps should be included in the end-to-end control strategy of **Figure 3**: freeze–thaw history is a process parameter with quality consequences, not a logistics detail.

## 7 Future perspectives

### 7.1 Process analytical technology, AI, and predictive control

Future expansion systems will need richer process data and better models. Multiomics, imaging, metabolite profiling, cytokine measurements, and functional assays can be combined with process parameters to predict product quality during manufacturing. An artificial intelligence-based integrative multiomics model has already been proposed for predicting T-cell quality during production (106). Such models should be developed cautiously. They require representative training data, external validation, interpretability, version control, and a clear relationship to critical quality attributes. Otherwise, an AI system may reproduce historical bias or make confident predictions outside its validated range.

Soft sensors are the enabling measurement layer for such control. Raman and dielectric (capacitance) spectroscopy are established in protein bioprocessing for estimating biomass and metabolic state and are now being evaluated for cell therapy; combined with mechanistic or data-driven models, they could estimate viable cell density, nutrient consumption, and metabolic shifts from inline signals without consuming cells. Their transfer to CAR-T manufacturing is not trivial: calibration must survive changes in donor material, medium formulation, and activation reagent, and sensor models must be validated for the specific process rather than borrowed from antibody platforms. Until such models are qualified, cell count, viability, and metabolite measurements will remain predominantly at-line, as reflected in **Figure 3**.

Model validation under GxP is the second requirement. An AI model used for process decisions should be managed as a validated software and analytical lifecycle: representative and versioned training data, predefined performance criteria, change control for model retraining, periodic revalidation, and human-readable decision logs. These requirements reinforce the near-term role of AI as decision support under human review, with autonomy earned incrementally as assay-to-outcome links are demonstrated (106).

We propose that, in the near term, the most realistic role for AI is decision support: detecting process drift, recommending feeding or harvest windows, identifying high-risk batches, and prioritizing confirmatory testing. Fully autonomous control will require validated links between sensor outputs, model predictions, and potency-relevant outcomes. Microfluidic profiling (83), rapid biophysical measurements (36), and miniaturized culture systems (82) could supply the high-frequency data needed for these models if they can be made robust enough for GMP use.

### 7.2 Decentralized and point-of-care production

Point-of-care and decentralized manufacturing seek to bring production closer to patients. Academic-center experience with closed semi-automatic bioreactors and closed production platforms shows that local CAR-T manufacture is technically feasible (107, 108). Multi-site place-of-care manufacturing has produced high remission rates in B-cell malignancies (109), and real-world experience from India demonstrates both the promise and the cost complexity of decentralized Prodigy-based production (110).

The decentralized model will succeed only if quality can be standardized across sites. Closed systems help, but they do not remove the need for trained personnel, validated facilities, raw-material control, release testing, deviation management, and regulatory oversight. Digital batch records, remote review, standardized consumable kits, and centralized model validation may make distributed production more practical. The long-term objective should be a network in which local manufacturing is locally responsive but centrally standardized.

### 7.3 Designing the next generation of consumables

The next generation of consumables should be designed around biological function rather than only physical containment. Culture bags could incorporate more consistent gas-transfer properties, integrated sensors, low-leachable contact layers, and sampling interfaces that preserve closure. Activation materials could combine defined ligand presentation with easy clearance and tunable mechanical properties. Coatings could be recombinant, xeno-free, stable after sterilization, and supplied with quantitative activity assays. Media and cytokine kits could be formulated for specific phenotypic targets rather than generic maximal growth.

Material innovation will matter most when it is paired with process evidence. A nanoparticle activation reagent (62), viscoelastic scaffold (46), or hydrogel microtube (65) should be evaluated against conventional bead-based activation not only for expansion but also for memory phenotype, exhaustion, potency, recovery, residual clearance, lot consistency, and cost per successful dose. This integrated evidence package is what will allow promising materials to move from proof of concept to routine manufacturing.

### 7.4 Toward interoperable closed platforms

The future device landscape is likely to include both highly integrated instruments and modular systems. Integrated instruments are attractive for standardized autologous workflows, while modular systems may better accommodate process innovation and diverse product types. In both cases, the field needs common definitions for closed operation, acceptable sensor performance, data integrity, consumable compatibility, and process-transfer evidence. Standardization should not freeze innovation; it should make innovation easier to validate and compare.

We envision that the most credible path forward combines QbD, single-use engineering, defined raw materials, and adaptive analytics. Closed-loop architectures such as the one shown in **Figure 3** will become useful when sensors and control algorithms are tied to meaningful cellular attributes. Rapid manufacturing will coexist with conventional expansion because different products and patient populations will have different optimal process designs. Decentralized production will expand only where quality systems can be replicated reliably. The field should therefore move away from asking which single device is best and toward asking which combination of biology, materials, equipment, and control strategy reproducibly delivers a defined therapeutic product.

## 8 Conclusions

Ex vivo T-cell expansion sits at the intersection of immunology, materials science, bioprocess engineering, and regulatory science. Its purpose, as developed in Sections 1 and 6.1, is to reproducibly manufacture a therapeutically defined population rather than to maximize cell number. Static gas-permeable systems, rocking-motion bioreactors, stirred tanks, hollow-fiber devices, and emerging three-dimensional or microfluidic platforms each offer distinct advantages, but none removes the need to understand how culture conditions shape T-cell fate.

Consumables should be viewed as active process components. Activation reagents, coatings, films, connectors, sensors, and single-use assemblies influence activation, gas exchange, sterility, traceability, and cost. Integrated automated systems can reduce open handling and operator dependence, but their value will be limited until monitoring, potency prediction, interoperability, and validation standards improve. Ultimately, the next decade of CAR-T manufacturing will be defined not by the isolated brilliance of a single device, but by the intelligent integration of biological insight, functional materials, closed-system design, and data-driven control—a convergence that promises to transform CAR-T therapy from a bespoke artisan craft into a robust, reproducible, and broadly accessible therapeutic platform.
